# Two-shift operation mode can improve the efficiency and comfort of flexible ureteroscopic holmium laser lithotripsy for the treatment of renal calculi larger than 1.5cm

**DOI:** 10.1590/S1677-5538.IBJU.2019.0219

**Published:** 2019-12-17

**Authors:** Sun Hui, Yang Qingya, Yin Xinbao, Liu Ming, Li Gonghui, Chen Jun

**Affiliations:** 1 Qilu Hospital of Shandong University, Qingdao, China;; 2 Sir Run Run Shaw Hospital of Zhejiang University, Hangzhou, China

**Keywords:** Ureteroscopy, Lithotripsy, Laser, Kidney Calculi

## Abstract

**Purpose:**

To compare two-shift operation mode and single player mode different impact on surgical results and operator comfort in flexible ureteroscopic holmium laser lithotripsy for renal calculi larger than 1.5cm.

**Materials and Methods:**

From december 2017 to december 2018, 92 patients with renal calculi admitted to Qilu Hospital and were treated through flexible ureteroscopy. They were randomized in two-shift group (n=50) and single player group (n=42). The operative time, blood loss, hospitalization stay after operation, residual fragments (≥4mm) rate, fragmentation speed, postoperative complications and operator's fatigue score were compared.

**Results:**

There was no significant difference between two groups regarding age, gender, illness side, stone size, blood loss, operative time, postoperative hospitalization stay, complications, etc (p >0.05). The fragmentation speed was 44.5±20.0mm3/min in two-shift group compared with 34.2±17.3mm3/min in single player group (p=0.037). Residual fragments (≥4mm) rate after first surgery was 18% in two-shift group, while the residual fragments (≥4mm) rate was 40.5% after first surgery in single player group (p=0.017). The total fatigue score of two-shift group was 8.4 compared to 29.9 in single player group (p <0.001).

**Conclusion:**

In flexible ureteroscopic holmium laser lithotripsy for the treatment of renal calculi larger than 1.5cm, two-shift operation mode can raise the fragmentation speed and stone clearance rate, as well as significantly lower operator's fatigue level and improve operator's comfort.

## INTRODUCTION

Urinary tract calculi is a common disease and its incidence is 5∼15% (1) worldwide, 1-5% (2) in our country. With significant improvement in the armamentarium, flexible ureteroscopy (FURS) evolved rapidly over the last decade and had become a viable alternative to extracorporeal shock-wave lithotripsy and percutaneous nephrolithotomy (PCNL), even for larger renal calculi (3–8). With the expansion of indication, flexible ureteroscopy has been used for the treatment of renal calculi larger than 1.5cm. However, holmium laser lithotripsy is a time-consuming process, the increasing volume and complexity of renal calculi would inevitably lead to operative time increase. During the operation, the surgeon has to hold the endoscope and focus on the target all time in a standing position. The suboptimal ergonomic posture may result in orthopaedic complaints (9, 10). Classic FURS usually needs single surgeon to complete the lithotripsy process. Two-shift operation mode for lithotripsy in FURS was rarely reported. Based on this belief, our study purposed to compare two-shift operation mode with classic single player mode on the impact to surgical results and operator comfort in flexible ureteroscopic holmium laser lithotripsy for renal calculi.

## MATERIALS AND METHODS

### Patients

A total of 92 patients with renal calculi (>1.5cm) were enrolled in our study from December 2017 to December 2018. Inclusion criteria consisted of the diameter of renal calculi larger than 1.5cm. The patients with acute urinary tract infection and severe urinary tract malformation were excluded. In order to pre-expand the ureter, all patients placed a double J tube 2 weeks before surgery. We used random number function for the sample randomization before study. 92 patients were randomly divided into two groups: one group required two surgeons to operate (Two-shift Group) and the other group required one surgeon to participate in the lithotripsy procedure (Single-Player Group). This study was conducted in accordance with the declaration of Helsinki. This study was conducted with approval from the Ethics Committee of Qilu Hospital. Written informed consent was obtained from all participants.

### Surgical setting and procedure

1. OLYMPUS^™^ URF-P5: tip diameter 7.2Fr, bent angle-180∼275°;
2. COOK^™^ access sheath: diameter 10/12Fr, 45cm for male, 35cm for female;
3. LUMENIS^™^ Power Suite 60W: laser fiber Diameter 200um.

Two surgeons who had received systematic training and had more than 50 cases of individual surgical experience of FURS performed the treatment. Two-shift group required two surgeons to participate in the lithotripsy procedure and the participants took turns to operate, 15 minutes per person each time. Single-player group required just one surgeon of the medical team to participate in the lithotripsy procedure and lithotripsy procedure was finished by the same surgeon from beginning to the end. The procedures were performed under general anesthesia. Both groups were asked to adopt the”dusting settings”approach with holmium laser setting: energy 0.4-1.0 J and frequency 25-30 Hz (low-energy and high-frequency). The laser energy was adjusted according to the hardness of the stone and the reaction to the laser. No stone basket was used during surgery. After the operation, the surgeon was requested to complete the ergonomics questionnaire immediately.

### Clinical evaluation and Statistics

Observation and comparison points of our study included operative time, blood loss, hospitalization stay after operation, stone free rate, fragmentation speed (cubic millimeter per minute), postoperative complications and operator's fatigue score. The first follow-up was one month later and before remove of Double-J stent. At first follow-up, Computed Tomography (CT) was performed. If the residual stone diameter was less than 4mm, it was considered as clinically insignificant residual fragments. If the patient's residual stone diameter was greater than or equal to 4mm after first surgery, they would be recommended for further treatment. The measure of stone volume was based on preoperative computed tomography by using AW VolumeShare^™^ (GE, Fairfield, Conn, USA). Operator's fatigue score was based on an ergonomics questionnaire referred to Chinese Ophthalmology and a previously published questionnaire (11, 12). Numerical data were expressed as mean with standard deviation (SD). Statistical analysis was performed with SPSS v.16 (IBM Corp, Armonk, NY, USA) including chi-square test for categorical variables and independent T test for numeric variables. P values <0.05 were considered statistically significant.

## RESULTS

### Preoperative clinical data

There were in total 92 cases which were randomized in two-shift group and single-player group. 50 cases were randomized in two-shift group (male 29, female 21) and mean age was 50.0±11.8years. Meanwhile, 42 cases were randomized in single-player group (male 30, female 12) and mean age was 49.6±10.7years. There was no differences between two group's preoperative data (including age, gender, ill side, lower calyx number, stone size and volume; p >0.05). More details are shown in Table-1.

**Table 1:** Comparison of two group's patient data.

Criteria	Two-shift group	Single-player group	Comment
Age, year	50.0±11.8	49.6±10.7	–
Male/female	29/21	30/12	–
Left/right	28/22	25/17	–
Lower calyx/all	26/50	26/42	–
Stone size, mm	21.0±5.1	21.6±5.9	Multiple calculi: sum of lengths
Stone volume, mm3	2759.9±1307.6	2717.9±1237.2	Calculated based on preoperative computed tomography

### Clinical experience

All operations were successfully performed. Details of clinical experience are shown in Table-2. No difference was shown regarding the operative time, blood loss, complications and hospitalization stay after operation (p >0.05). With respect to residual fragments(≥4mm) rate of first-surgery two-shift group (18%) revealed statistically significant differences with single-player group (40.5%) (p=0.017). Fragmentation speed also favored two-shift group (mean: 44.5±20.0mm^3^/min) over single-player group (mean: 34.2±17.3mm^3^/min) and reached statistical significance (p=0.037). Ureteral stone street forming occurred in two cases of two-shift group and they recovered after ESWL. One case of single-player group bled in prostate and two cases had postoperative fever. Others recovered well with no significant complication.

**Table 2:** Comparison of two group's surgery results.

Criteria	Two-shift group	Single-player group	Comment
operative time (min)	84.7±32.4	90.6±24.5	–
blood loss (mL)	10.4±9.2	8.1±5.7	–
hospitalization stay (d)	2.2±0.6	2.5±1.4	–
residual fragments (≥4mm) rate (%)	18.0	40.5	–
fragmentation speed (mm^3^/min)	44.5±20.0	34.2±17.3	just for patients without clinically significant residual fragments
complications	2	3	fever, stone street forming and prostate bleeding

### Evaluation of Ergonomics

Two group's fatigue score are shown in Table-3. There was a significant difference when comparing the fatigue score of two-shift group and single-player group (total score: 8.4 vs. 29.9; p <0.001), and the difference was most obvious concerning eye drying, musculoskeletal pain, forearm pain, elbow and wrist stiffness, finger numbness and leg pain.

**Table 3:** Comparison of two group's fatigue score concerning the ergonomics.

No. of complaints (0-5)*	Two-shift group	Single-player group
eye ache	0.9 (0-3)	2.0 (0-4)
eye bulge	0.4 (0-2)	1.8 (0-3)
eye blur	0.2 (0-1)	0.8 (0-2)
eye drying	0.7 (0-2)	2.0 (0-4)
photophobia	0.2 (0-1)	1.2 (0-3)
Musculoskeletal pain	1.0 (0-3)	2.6 (1-5)
Neck pain	0.3 (0-1)	1.3 (0-3)
Shoulder stiffness	0.3 (0-2)	1.7 (0-4)
Arm pain	0.4 (0-2)	1.9 (0-4)
Forearm pain	0.9 (0-2)	2.8 (0-5)
Elbow stiffness	0.4 (0-1)	2.0 (0-4)
Wrist stiffness	0.9 (0-2)	3.0 (0-5)
Finger numbness	0.5 (0-2)	2.2 (0-4)
Back pain	0.3 (0-1)	1.5 (0-4)
Leg pain	1.0 (0-3)	3.1 (0-5)
**Total score**	**8.4 (0-26)**	**29.9 (2-49)**

* 0 = no complaints; 1 = little pain; 5 = severe pain.

## DISCUSSION

With significant improvements in the armamentarium, FURS is playing an important role in the management of nephrolithiasis. The advantages of flexible ureteroscopy are indisputable (13) which include minimum noninvasive surgical trauma, low complication rate, short hospital stays and repeatable ongoing treatment. These merits lead to more and more surgeons and patients to select FURS for treatment of renal calculi. As operative experience accumulate and treatment expectations of patients become greater, the indications of FURS for renal calculi have expanded to even larger than 2cm (14–17). With the expansion of the FUR'S indications, FURS has been used for the treatment of larger and more complex calculus. Pre-stenting improves the stone free rate (SFR) and reduces intra-operative complications (18). Pre-stenting positively affects safety and efficacy of URS (19). Therefore, routine pre-stenting was required in our study. According to our experience, pre-stenting can facilitate access of the ureteroscope and sheath. However, holmium laser lithotripsy is a relatively time-consuming process, with the volume and structure complexity of stones increasing, extension of fragment time will be inevitable.

During FURS, the surgeon is used to insert and advance laser fibre, and control irrigation while holding the endoscope. Through foot pedal, operators usually activate holmium laser lithotripsy in a standing position and the procedure of lithotripsy requires surgeon continuous focusing on the target. Long-time maintaining this position will inevitably lead to operator's discomfort and may also have a negative impact on performance of FURS. The fatigue problem can be overcome by teamwork. As shown by the questionnaire comparing the ergonomics of two shift operation and single-player operation in FURS (Table-3), two shift work mode provides a suitable solution to improve ergonomics significantly (total score: 8.4 vs. 29.9). Meanwhile, two shift operation proved to be faster for lithotripsy than single-player operation, reflected by higher fragment speed (44.5±20.0mm^3^/min vs. 34.2±17.3mm^3^/min). And the residual fragments (≥4mm) rate after first operation of two shift group was also lower than single-player group (18.0% vs. 40.5%). The residual stone rate of two groups in our study was high, and this result might be related to the “dusting and no basketing” strategy we adopted. Recent data comparing laser lithotripsy with dusting versus basketing suggest higher rates of residual fragments with dusting but less utilization of ureteral access sheaths and potentially shorter operative times (20).

However, we think that the operator's discomfort may be another reason for imperfect performance of FURS resulting in the higher residual fragments (≥4mm) rate and lower fragment speed. As the process of lithotripsy is tedious and even boring, long term uncomfortable positions often wear down operator's patience and energy. This status of exhaustion also results in lower fragmentation speed. For the treatment of special types of stones, such as infection stones, operative time should be controlled as short as possible, in order to reduce the incidence of postoperative sepsis. In these cases, how to improve the fragment speed and reduce operative time is considered particularly important. Therefore, two shift operation mode can be a good solution for this problem.

With improvements in the armamentarium, FURS will provide more superior image quality. Saglam et al. (21) reported robotic flexible ureteroscopy has the advantage of significant improvement of ergonomics compared to classic flexible ureteroscopy. Even though, two shift mode can be a recommended approach for the treatment of renal calculi in FURS.

## CONCLUSIONS

In flexible ureteroscopy for the treatment of renal calculi larger than 1.5cm, two-shift operation mode can raise the fragmentation speed and stone clearance rate, as well as significantly lower operators' fatigue level and improve operator's comfort.
